# Experience in the Use of Nitrous Oxide and Oxygen with Rebreathing in Military Surgery

**Published:** 1918-04

**Authors:** 


**_Experience in the Use of Nitrous Oxide and Oxygen with Rebreathing in Military Surgery._** By Elmund G. Boyle. Capt., M. R. C. S. Abs. from the *Lancet*, Nov. 3, 1917.

B. gives the patient a preliminary injection of morphine, atropine, and scopolamine about half an hour before the operation[1]. He uses the Gwathmey apparatus with $N_2O$ and O cylinders in administering the anesthetic.

The writer insists that the room should be perfectly quiet during the induction. Although Gwathmey prefers that the patient's head should not be raised by a pillow, B. finds it better for the comfot of the patient to allow the head to be raised during the induction, and lowered when the patient is unconscious. It is sometimes well to insert a dental prop between the teeth, especially if the patient does not breathe freely through the nose.

The bag is three-parts filled with $N_2O$ and O in the proportion of 4 to 1. The face piece must fit accurately. The valve at the top of the face piece is kept open for a few breaths and then turned off so that rebreathing starts almost from the beginning. The supply of nitrous oxide is then increased to lessen the period of induction. If cyanosis is observed, a little oxygen should at once be added. As soon as anesthesia is produced, the original proportions of $N_2O$ and O should be restored. If vomiting occurs both $N_2O$ and O are increased and the patient carefully watched. The author prefers to deepen the anesthesia with ether rather than increase the $N_2O$. Although a certain amount of rebreathing, as Gwathmey states, is necessary for an even anesthesia, it should be noted that excessive rebreathing leads to vomiting.

The symptoms of an overdose of $N_2O$, according to Gwathmey, are stertorous breathing and excessive secretion of mucus. If this condition is allowed to go too far, it may become dangerous. The mask should quickly be removed, and artificial respiration should be started together with the administration of oxygen. The airway should always be kept clear.

Patients come out of the anesthesia quickly, and are conscious within two or three minutes. They are not generally sick. There is no appreciable rise in temperature in most cases. The gases are non-toxic and are therefore especially desirable in serious cases of sepsis.

This method, however, is contra-indicated if *absolute* relaxation

1. Morphia tartrate 1/6 gr.; atropine sulphate 1/120 gr.; scopolamine 1/100 gr. This is made up so that 5 minims is the dose. One drop of pure carbolic is added to 4 oz. of the mixture to prevent it from becoming flocculent.

is required, as in some abdominal cases and gall bladder operations.

The author has found that 200 gallons of $N_2O$ and 60 gallons of O are sufficient for 2 1/2 hours.

The writer warns against the dangers that may arise if this method is entrusted to persons who are careless and inexpert.

